# African Sorghum-Based Fermented Foods: Past, Current and Future Prospects

**DOI:** 10.3390/nu12041111

**Published:** 2020-04-16

**Authors:** Oluwafemi Ayodeji Adebo

**Affiliations:** Department of Biotechnology and Food Technology, Faculty of Science, University of Johannesburg (Doornfontein Campus), P.O. Box 17011 Johannesburg, Gauteng 2028, South Africa; oadebo@uj.ac.za; Tel.: +27-11-559-6261

**Keywords:** sorghum, fermentation, lactic acid bacteria, fermented products, food security, 4th industrial revolution (4IR)

## Abstract

Sorghum (*Sorghum bicolor*) is a well-known drought and climate resistant crop with vast food use for the inhabitants of Africa and other developing countries. The importance of this crop is well reflected in its embedded benefits and use as a staple food, with fermentation playing a significant role in transforming this crop into an edible form. Although the majority of these fermented food products evolve from ethnic groups and rural communities, industrialization and the application of improved food processing techniques have led to the commercial success and viability of derived products. While some of these sorghum-based fermented food products still continue to bask in this success, much more still needs to be done to further explore evolving techniques, technologies and processes. The addition of other affordable nutrient sources in sorghum-based fermented foods is equally important, as this will effectively augment the intake of a nutritionally balanced product.

## 1. Introduction

In terms of production quantity, sorghum is the fifth most important cereal crop in the world after rice, wheat, maize and barley, and the most grown cereal in Sub-Saharan Africa, after maize [1,2,3]. It remains one of the most versatile cereal crops on the continent, serving as a staple and main meal for millions of people [1,3,4]. It is an important source of calories, variety of nutrients and beneficial food components [5,6,7]. With the increasing world population, decrease in water supply and the effects of climate change, this drought resistant food crop is vital for human utilization and will be an important crop for the future.

Amongst all the available food processing techniques, fermentation is an age-long process, known to improve nutritional qualities, palatability and consumer appeal [8,9,10,11]. Derived fermented food products continue to constitute an important part of our daily diet and are estimated to provide about a third of world food supplies [12]. These foods are known to confer beneficial effects, including therapeutic and functional properties, in addition to possessing antimicrobial, antioxidant, probiotic and cholesterol-lowering attributes, and are a source of some other important bioactive compounds [11,13,14,15,16,17]. Accordingly, fermented sorghum-based foods have a long history and strong cultural ties to the African people in particular.

Although sorghum is the third most produced cereal grain in Africa after maize and rice, it has not been fully utilized for industrial processing as compared to other major cereals. Rapid urbanization, an increasing population, the cost of other imported cereal commodities and the demand for high quality functional foods have nonetheless driven the rise in the consumption of sorghum-based food products. Further to this, the demand for gluten-free foods for people with celiac disease and other intolerances to wheatpositions sorghum as a suitable substrate for these types of diet. There is also an upsurge and renewed interest in the use of alternative, climate-smart and traditional grains, like sorghum, in modern food products [18]. Particularly important is the vital role that sorghum plays as one of the main sources of food and energy, similarly to other cereals. This review describes sorghum-based fermented foods, highlighting notable traditionally processed products that have become a commercial success. Prospects for the future and possibilities of areas that should be explored are also emphasized.

## 2. Overview on Sorghum

Judging from the 2017 available data from FAOSTAT, Africa is the largest contributor to world sorghum production, with a production quantity of approximately 29.7 million tonnes [1]. Sorghum grains are a widely adaptable species and mostly cultivated in tropical, subtropical and temperate regions [19]. As a drought tolerant and a climate-smart crop under the prevailing realities of climate change, its utilization is spread across diverse industries, including for animal feed, biofuels, forage, ethanol production and fodder preservation [20,21]. It remains one of the most versatile food crops in Africa.

Sorghum belongs to the Andropogoneae tribe and *Poaceae* family and is a known C_4_ crop (i.e., it uses the C_4_ carbon fixation pathway to increase its photosynthetic efficiency), particularly adapted to hot, drought-prone and semi-arid tropical environments with less rainfall. It is said to have originated from the Northeast quadrant of Africa [22]. Millet, barley, teff and wheat are also members of the *Poaceae* family [23] and are likewise known for their ecological dominance in many ecosystems, as well as their capacity to grow in low rainfall and harsh environmental extremes conditions [24]. As indicated by Ratnavathi and Komala [25], over 20 sorghum species are known and these include: *Sorghum almum, Sorghum amplum, Sorghum angustum, Sorghum arundinaceum, Sorghum bicolor, Sorghum brachypodum, Sorghum bulbosum, Sorghum burmahicum, Sorghum ecarinatum, Sorghum exstans, Sorghum grande, Sorghum halepense, Sorghum interjectum, Sorghum intrans, Sorghum laxiflorum, Sorghum leiocladum, Sorghum macrospermum, Sorghum matarankense, Sorghum nitidum, Sorghum plumosum, Sorghum propinquum, Sorghum purpureosericeum, Sorghum stipoideum, Sorghum timorense, Sorghum trichocladum, Sorghum versicolor, Sorghum verticiliflorum* and *Sorghum vulgare var. technicum*. Notable among these is *S. bicolor*, known for its food use.

**Figure 1:**
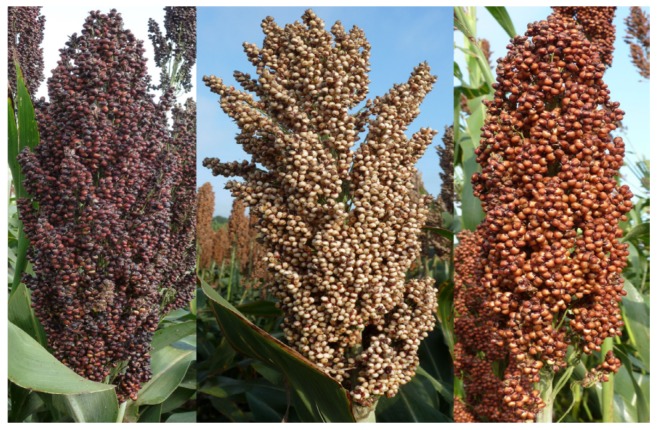
Mature panicles of different types of sorghum in the field. Black (left), white (middle), and red (right) sorghum (Adapted from Awika [27]).

Sorghum grains are single seeded, with their pericarp surrounding and tight adherence to the seed coat [6,26]. Its grass varies between 0 and 6 m in height, with deep, spreading roots and a solid stem. Sorghum kernels are usually flattened spheres measuring about 4, 2.5 and 3.5 mm in length, thickness and width, respectively, with an average weight of about 25 mg [26]. Sorghum grains can typically be white, pale orange, tan, red, dark brown and brownish-red [5,26,27], but the major commercially available ones are the black, white and red (Figure 1). The color of the testa (seed coat or pericarp) are genetic characters controlled by the R and Y genes [22,28].

### 2.1. Nutitional Composition of Sorghum

In developing countries, especially in Africa, over 78% of the sorghum produced is used for food, with about 14% for animal feeding and 7% for other uses [29]. Extensive studies on the composition of sorghum have indicated that the grain is a good source of energy, carbohydrates, polyunsaturated fatty acids (PUFAs), minerals, vitamins and some essential amino acids [27,28,30]. Proximate composition of sorghum from earlier studies has indicated that its protein content ranges from 6.2% to 14.9%, carbohydrates (54.6%–85.2%), fat (1.3%–10.5%), ash (0.9%–4.2%) and fibre (1.4%–26.1%) (Table 1). The variations in these values might possibly be related to the genotypes of the grains and growth conditions, as well as other cultivar specific differences. As indicated by previous authors [27,31], starch, including dietary fiber derived from cellulosic cell wall carbohydrates, is a major component of sorghum, constituting about 75% of the grain. The presence of non-starch polysaccharides (NSPs) in sorghum grains could be suggestive of their potential ability to improve bowel function and lower cholesterol levels [32,33].

Sorghum contains both some of the essential and non-essential amino acids, including alanine (7.34–9.62 g/100 g), aspartic acid (4.83–7.06 g/100 g), glutamic acid (17.5–28.12 g/100 g), leucine (12.02–14.48 g/100 g), phenyalanine (4.03–5.62 g/100 g), proline (6.66–12.34 g/100 g) and valine (4.22–6.86 g/100 g) (Table 2), but limited in lysine and tryptophan. It does, however, have beneficial bioactive peptides and protein fractions, including 2-kDa antiviral peptide, α-kafirin, karifin, protease, amylase and xylanase inhibitors, as well as cationic peroxidase, which exerts anticancer, antiviral, antioxidant, cholesterol-lowering and antihypertensive effects [46,47,48,49].

Available studies have also indicated that sorghum contain minerals (Table 3) and vitamins (Table 4), both of which constitute part the essential nutrients required by humans to perform the functions necessary to sustain life. Sorghum contains fairly high levels of potassium (K) (900–6957.67 mg/kg), and phosphorus (P) (1498–3787.25 mg/kg), minerals known to assist with muscle movement, keeping the nervous system healthy and building strong bones and teeth. Vital vitamins reported in sorghum also include the B-vitamins (0.1–19.9 mg/100 g), vitamin E (1.38 mg/100 g). Trace amounts (maximum of 0.01 mg/100 g) of β-carotene (a vitamin and precursor of vitamin A) have also been reported [50,51], an indication that sorghum cannot be considered a good source of β-carotene and vitamin A. Considering the nutrient deficiencies in under-developed and developing countries in Africa, the limitation of some of these vital nutrients could be addressed by complementing sorghum with legumes (a plant source), as well as animal products. This will contribute to ensuring a nutritional balance of these nutrients, and assist in alleviating and counteracting micronutrient deficiencies.

### 2.2. Bioactive Constituents of Sorghum

Sorghum grains and its subsequent food products are excellent sources of health promoting constituents including polyphenols, bioactive lipids, policosanols, phytosterols [62,63,64,65] and starch/carbohydrate fractions [66,67]. Numerous other properties of sorghum indicate its potential as a health food, including the absence of gluten (recommended for celiac and gluten celiac patients) and a relatively low glycemic index and load, thus reducing the risk of diabetes. The role of sorghum in lowering low-density lipoprotein (LDL), has also been reported, as well as its steroid-binding properties and its role in combating arthritis and rheumatism [65,68,69].

The most investigated bioactive component in sorghum are the polyphenols, which, in part, is due to the diversity of these compounds in sorghum grain. According to Awika [27] and Girard & Awika [70], sorghum is the most diverse cereal in terms of the amounts and types of polyphenols present in them. It has one of the widest ranges of health beneficial components compared to other cereals [70]. The phenolic compounds include flavonoids, phenolic acids and condensed tannins (unique to few cereal grains) [17,27,70]. These condensed tannins (located in sorghum testa and pericarp) protect the seed against pest invasion, fungi, birds and other rodents [29]. Although tannins in sorghum can be considered desirable from an agronomic perspective, together with dhurrin (a cyanogenic glucoside in located mainly in the aerial shoot and sprouted seeds), they are considered the two major anti-nutritional factors (ANFs) in sorghum [29,71]. While tannin decreases starch and protein digestibility, cause dysfunction of cellular membranes and cause dysfunction of cellular membranes [29,70,72,73], dhurrin could cause cyanide poisoning (respiratory difficulty, nausea, abdominal distension [71,74]. Several processing methods, including fermentation, have, however, been reported to reduce these ANFs, improve digestibility and release bound nutrients and health beneficial components [11,29,70,75,76,77,78,79].

Dietary polyphenols in sorghum are reported to show high-antioxidant capacity when compared to other grains such as rice, millet, maize and wheat [80,81,82,83], which has been attributed to the redox chemistry of sorghum polyphenols [27]. Other beneficial effects of sorghum and its derived products include their ability to improve glycemic response, prevent cancer and confer anti-inflammatory effects. Other studies in the literature have also reported that sorghum phenolic extracts exert a protective effect to help prevent the onset of neurodegenerative related diseases, confer antidiabetic and anticancer effects, reduce swelling (oedema) and lower the incidence of oesophageal cancer [83,84,85,86,87,88,89]. Evidence for this has been demonstrated in previous studies and recently summarized in the reviews of Girard & Awika [70] and Aruna & Visarada [65]. This might also explain the interest and continued research into the role and effect of these compounds in sorghum and its exploration for mitigation of human health diseases. A viable route of ensuring that these benefits extend to humans would be through the incorporation of sorghum into diet. This could be through the appropriate transformation of sorghum grains into various other beneficial food forms, which would ensure possibility of obtaining various value-added food products.

## 3. Sorghum Fermentation

While numerous techniques are available for the transformation of sorghum into other food forms, fermentation is still regarded as one of the oldest means of processing sorghum, and remains largely significant because of the beneficial functionalities it confers on foods. Fermentation can simply be defined as the intentional conversion/modification of a substrate into new products/forms, through microbial actions. This biochemical process is usually done with the goal of obtaining a specific product. As such, numerous changes occur during fermentation, leading to the modification of the sorghum substrate and production of metabolites. Such alterations influence taste, appearance, texture, color, flavor, shelf life and nutritional properties of derived products.

As with other fermentation processes, sorghum fermentation is generally classified into three categories viz.: spontaneous (also referred to as wild or natural), backslopping and controlled fermentation [11,90]. Spontaneous fermentation has been done for many years and basically involves the addition of water to sorghum and incubation of the mixture under suitable conditions of temperature and desired time. Fermentation during this process is usually through the sequential and competitive action of a plethora of microorganisms, with the best adapted strain(s), having a better growth rate, eventually dominating the microbiota. Microorganisms involved mainly come from the seed surface, and the subsequent changes are usually due to enzymatic activity exerted by these surface microorganisms, as well as endogenous enzymes in the grain. Occasional failure, slow fermentation rates, variation in qualities and lesser acidification probably led to a better craftmanship and the birth of backslopping [90,91], which involves a re-innoculation of a previous successful fermentation batch into a new process, a procedure which guarantees a better fermentation process.

Advances in fermentation technologies and the increasing demand for fermented products of better and consistent quality have led to the use of starter cultures for a more controlled fermentation process [91,92]. This has necessitated the selection and identification of specific strains (starter cultures), with high competitiveness and shorter lag phases [93] and subsequent use of such organisms in controlled fermentation process. As such, various studies over the years have investigated the dominant strains in sorghum fermented foods and subsequently isolated, purified, characterized and preserved these microorganisms with the objective of using them to obtain final fermented products with the desired characteristics.

Generally, in sorghum, lactic acid bacteria (LAB) are the most dominant microorganisms during fermentation (Table 5), with lesser occurrence and reports of yeasts and fungi [91,93,94,95]. According to Teusink and Molenaar [96], environments in which LABs thrive are rich in proteins, sugars, vitamins, nucleotides and fats, and this could explain their predominance in sorghum microflora. It is also related to their high acidic tolerance and relative superiority in the utilization of starchy sorghum substrates, as well as the versatile carbohydrate metabolism thereof. According to Gänzle [97], this group of microorganisms are exploitative competitors, and inhibit other microorganisms through rapid utilization of abundant carbohydrates and accumulation of acetic and lactic acids. It is thus unsurprising that lactic acid fermentation is the most common form of sorghum fermentation type and mainly carried out by LABs (Table 5). LABs are generally recognized to be safe and beneficial, with some strains having health-promoting (probiotic) features. These group of microorganisms reduce the risk of fermentation failure and the fermentation period, while improving the value of the end product, as they have the ability to synthesize organic acids, inhibit food poisoning and spoilage bacteria through their antimicrobial, bactericidal and bacteriostatic effects [84,91,98]. The presence of other microorganisms is noteworthy, and they could have possibly participated in the fermentation process and/or are opportunistic microorganisms in the fermentation process. According to Capozzi [90], a broad diversity of microorganisms is associated with variety of raw materials used, fermentative behavior and nature of obtained products, and as such the multiplication and presence of these other undesired microorganisms might be difficult to control/limit in natural (spontaneous) fermentation.

**Figure 2:**
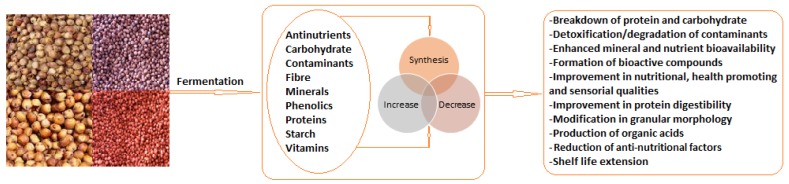
Modifications to sorghum during fermentation.

Sorghum fermentation usually results in changes to and the subsequent improvement of nutritional qualities, taste, shelf life, aroma and structural modification. Similar to other cereal fermentation processes, fermentation of sorghum leads to a modification (increase/decrease) of inherent metabolites and constituents (Figure 2), activation of enzymes, decrease in pH levels, increased metabolic activities and microbial actions with a consequent decrease in ANFs, detoxification and degradation of contaminants [9,11,17,145,146,147,148,149,150,151,152,153].

These modifications are, in part, due to proteolysis, with the possible formation of monomers from large molecules. Fermentation also leads to the production of enzymes that trigger the breakdown of substrates, improving the nutritional quality [154]. The production of organic acids, leading to a decrease in pH and a corresponding rise in titratable acidity, with accompanying changes in functional properties (such as emulsifying and oil- and water-binding capacity) have also been reported in the literature [4,17,146,155].

In addition to these modifications, fermentation equally affects the amount and composition of phenolic compounds. The metabolism of phenolic compounds and an increase/decrease in antioxidant activities during fermentation have been extensively reported [11,17,91,156,157,158,159,160]. Through the metabolic activities of microbes, fermentation also induces the structural breakdown of the cell wall, leading to the synthesis of various bioactive compounds. Equally important are the roles of amylases, proteases and xylanases derived from the fermenting microorganisms, and the cereal grain that contributes to the modification of the grain and the distortion of the chemical bonds, consequently releasing bound phenolics [11]. During fermentation, these phenolic compounds are metabolized and modified by the fermenting organism into other conjugates, glucosides and/or related forms through decarboxylation and hydrolysis, as well as esterification [11,159,160].

Not only do the metabolites produced inhibit the growth of pathogenic and spoilage microorganisms, they equally suppress them. Beyond its preservative effects, fermentation improves the palatability, nutritional profile and effects desirable organoleptic characteristics that impart the desired flavor, texture and aroma. Other benefits in addition to this are the extension of shelf life and the production of health beneficial constituents. The microbiology and biochemistry of the fermentation process have been well documented [9,161,162], with these studies indicating that the endogenous microorganisms that are activated during the natural fermentation process or the starter cultures used facilitate the subsequent activities and production of several compounds that result in the aforementioned changes.

## 4. African Sorghum Fermented Food Products: Traditional and Value Added Products

Traditionally, the sorghum fermentation process is usually carried out in small and household scales. These are characterized by the use of indigenous, non-sterile equipment under unhygienic conditions, with unattractive packaging for the derived products. Furthermore, the inconsistencies of the ingredients and innoculum lead to pH variation in the final products. Due to the socio-economic, nutritional and cultural role these fermented foods play in African communities and households, concerted efforts have been made and studies have been conducted in improving the fermentation process for the development of indigenous fermented foods. This section provides an appraisal of traditional fermented sorghum-based products and their development into shelf-stable value-added products on the market.

Beverages are most probably one of the most consumed sorghum-based fermented products known. Both alcoholic and non-alcoholic African sorghum beverages are known under a variety of names (Table 4). While the traditional processing of these products has been documented in the literature, intensified efforts towards their developments have led to their availability on the market. From being traditionally brewed in local pots and served in calabash and traditional utensils, recent trends have seen them become products with significant socio-economic impacts. Their fermentation processes have been industrialized, and some of these products now flood the market in attractive packages and are readily available.

In tandem with recent trends of functional foods, researchers in South Africa created a functional beverage called *niselo* by including probiotics in a sorghum beverage to ensure consumers derive benefits beyond the inherent basic nutrition. *Motoho*, a non-alcoholic beverage from *ting* has also been recently developed through a modern commercial process by fermenting the sorghum with a specific commercial lactic acid bacteria strain, and the subsequent addition of chemical preservatives and additives [18]. A prominent Southern African sorghum-based opaque beer that is also readily available on the market is *umqombothi*. Through concerted efforts, these products have been industrialized, packaged, commercialized and made available in different flavors across Southern Africa.

Other fermented sorghum products of commercial importance and prominence are West African *kunu* and Ugandan *bushera*, which have evolved from products developed with traditional fermentation processes using calabash under unhygienic conditions into shelf stable products. According to Rosentrater and Evers [163], porridges made from cereals such as sorghum are one of the most important dishes consumed by the inhabitants of Sub-Saharan Africa. Both thick and thin porridges are made basically differing in the flour/water ratio required and consumed across ethnic divides. Successful products in this regard, which stemmed from their indigenous form to become commercially viable products, are *uji*, a Kenyan gruel and *ogi-baba*, a sorghum-based fermented cereal pudding from Nigeria.

## 5. Safety of African Sorghum Fermented Foods

Irrespective of the region in the world, food safety issues remain critical for individuals, food businesses and the relevant authorities. Such safety concerns extend to that of fermented foods, despite the numerous advantages attributed to the consumption of these foods earlier highlighted herein. Reports of some studies have indicated the presence of opportunistic pathogens and/or their toxins in some African sorghum-based fermented foods (Table 6). An appropriate assessment of the risk associated with these reported pathogens should, however, be carefully considered, as cell counts and/or the frequency of their presence would indicate if they really are of concern in these foods.

Judging from numerous studies and reviews that have shown that fermentation and LABs are capable of reducing/degrading toxins and contaminants in foods [147,150,151,153,172,173,174,175,176], it could be postulated that the levels found could either be ‘residues’ and or ‘left-overs’ of the fermentation process. The role of post contamination of these products should also not be ruled out as a possible source of these opportunistic microorganisms. Challenges of safety also arise with spontaneously fermented sorghum-based foods. This is in part due to the broad diversity of microorganisms causing an “unhealthy” competition among the fermenting microbiota, leading to the production of toxic by-products that compromise the safety of the food. Although much more prevalent in rural communities, safety issues of derived fermented foods can be traced to all or either of the following: (i) raw materials; (ii) processing equipment, items and materials; (iii) storage conditions and packaging; and/or (iv) biological, physical and chemical contaminants through processors/handlers and the processing environment. Since safety challenges in sorghum-based fermented foods (similar to other fermented foods) usually come from all or some of the aforementioned routes [75,90,154], less contamination must be ensured in raw materials, whilst ensuring the sterility of processing equipment and all other items during the production of the fermented food. Equally important are hygienic conditions for the handling, packaging and storage of fermented foods, to mitigate against post-processing contamination.

Various scientific studies and evidences have demonstrated the crucial importance of starter cultures and other modern microbial biotechnological solutions in food fermentation to ensure food safety [90,91]. This desired effect has been attributed to various biological activities, including domination of the indigenous microflora, faster acidification, reduced fermentation time and the suppression of undesired microbial strains/species [177,178,179]. Over and above, while the contribution of fermentation to food safety is evident, it cannot eliminate all food related health risks and should thus not be considered a control measure.

## 6. Future Projections

Fermented foods, including sorghum-based fermented products, are of prominent significance to the economy, health and nutrition of Africa. The nutritional value and profile of sorghum needs to be improved using techniques like the development of novel sorghum lines in order to improve the digestibility of sorghum proteins and genetic modification for improved nutritional value [180,181]. While these propositions could be successful, it would be important for high sorghum eating populations to complement their diet with foods rich in an appropriate nutrient balance, such as vegetables, fruits, animal products and nutrient-dense plant sources such as legumes [182,183].

Innovations and improvements to ensure the sustainability of these products should thus be continuous. The wave of the fourth industrial revolution (4IR) cannot be overlooked, and its effective application in sorghum fermentation is still lacking. Strongly intertwined with 4IR are artificial intelligence (AI) and machine learning (ML), relatively new concepts, with current utilization and potential for solving numerous complex challenges. As with other fields, AI and ML have a huge role to play in fermentation, including but not limited to product development, ensuring safety, improvement in product efficiency and plant productivity. These techniques have been applied in other sorghum related studies: Kashiri et al. [184] utilized an artificial neural network (ANN) for simulating and predicting the soaking behavior of sorghum kernel as a function of temperature and time, while Kaliba et al. [185] estimated the propensity for and intensity of the adoption of improved sorghum varieties in Tanzania using deep learning techniques. Sebayang et al. [186] also adopted ANN to investigate the relationship between bioethanol production, enzymatic hydrolysis and fermentation. The authors were able to optimize bioethanol production from sorghum grains, and indicated the effectiveness of the approach in reducing cost, time and effort associated with experimental techniques [186]. Further detailed description, classification and use of these AI and ML techniques is available in the literature, and can be consulted for further reading [187,188,189,190,191]. The specific and potential immediate application of AI and ML to sorghum-based fermented foods include predictive product development and optimization of fermentation processes. As indicated by the Institute of Food Technologists (IFT) [192], with other fields having rapidly embraced these techniques, the food and beverage industry is still trying to catch up with its counterparts in other industries that are ahead of it, and thus it calls for increased efforts in this regard.

The advent of the “omics” technologies (metabolomics, genomics, metagenomics, transcriptomics, foodomics, volatilomics and proteomics) have also opened up possibilities of better understanding and broader perspectives into the quality of fermented foods [8,64,91,193,194,195,196]. These big data bioinformatic techniques have provided tools to control, monitor, modify or improve such products. They also assist in providing deeper insights into the underlying molecular mechanisms of fermented foods, the dynamics of the fermenting microbiota, metabolic interactions and functionalities, as well as nutraceutical potential of these group of foods. In the past, the microbiota of fermented foods has been characterized using culture dependent techniques, which only focuses on a group of microorganisms, with an assumption that these targeted microorganisms are responsible for the fermentation process. Metagenomics, a culture-independent technique involving the sequencing of all DNA extracted from a sample, changed the way food microbial ecology is studied [195,196,197]. By providing a broader understanding of the microbiota of fermented foods, this would effectively guide in the selection of starter cultures for an improved fermentation process. Likewise, metabolomics involves the analysis of metabolites within a sample interest to answer specific biological questions that would further provide insight or assist in the selection of strains, process, substrate [8,195,197]. Through these technologies, and the earlier aforementioned ones, new strains of starter cultures with desirable functionalities could be identified and purified for use in fermentation. Also, better understanding of the characteristics and functions of already established starter cultures of fermentative microorganisms could also be obtained.

Other areas that could be explored to improve sorghum utilization and contribute to food security include the use of whole sorghum grains for fermented foods, with the provision of desirable health benefits and beneficial compounds in subsequent products compared to products from refined grains [11,160,198,199]. Potential technologies that can equally be utilized include encapsulation to improve the delivery of desired compounds into food [200,201,202], the adoption of novel food technologies, such as high-pressure processing (HPP) [203,204], ohmic heating [205,206] and pulse electric field (PEF) [207], as well as other non-thermal food processing technologies [91,208,209].

## 7. Conclusions

Fermented foods comprise part of the identity of ethnic groups, are important in social traditions, cultural folklore and are also crucial from a health, nutritional and economic perspective. The current market for sorghum and subsequent fermented products is huge and still growing. Sorghum is a valuable grain, particularly due to its health beneficial constituents and its status as a practicable option for coeliac and gluten intolerant people (due to its absence of gluten). While sorghum is also good source of carbohydrates (starch) and energy, combining it (during the development of fermented foods) with other affordable nutrient-dense food sources would improve the nutritional quality of the resulting products and would assist in alleviating some of the malnutrition challenges in Africa. Challenges regarding the possible development of undesirable colors, odors and off-flavors might arise during the development of these products, but once addressed, the resultant food products could lead to the birth of new niche markets and also contribute to consumer health and wellness on the continent, as well as uplift an already ailing economy, when such products can thrive commercially. Diversity in the diet of African populations should nonetheless be emphasized, as a diverse diet would best ensure a wider range of health promoting compounds and much needed nutrient adequacy.

## Figures and Tables

**Table 1:** Proximate composition of sorghum.

Proximate composition (%)	Jones and Beckwith [34]	Okoh et al. [35]	Adebiyi et al. [36]	Shawrang et al. [37]	Shargie [38]	Udachan et al. [39]	Awadelkareem et al. [40]	Ndimba et al. [41]	Singh et al. [42]	Ape et al. [43]	Jimoh and Abdullahi [44]	Mohapatra et al. [45]
**Ash**	1.2–1.3	0.90–1.52	1.98	4.20	1.44	0.92–1.75	1.28–1.78	1.61–2.03	1.90	2.07	1.12–1.68	3.17
**CHO**	NR	71.80–85.20	72.41	NR	NR	70.65–76.20	72.44–77.28	NR	NR	76.51	65.15–76.28	71.95
**Fat**	3.1–3.4	1.38–4.50	3.35	6.9	3.32	2.30–2.80	2.84–3.02	2.37–2.75	3.30	3.10	5.12–10.54	4.70
**Fiber**	1.8–1.9	1.47–2.45	2.25	19.5	1.83	1.40–2.70	1.72–2.02	NR	1.7	2.86	1.65–7.94	2.76
**Moisture**	NR	NR	10.66	8.1–8.5	NR	8.10–9.99	6.67–7.29	8.95–11.16	9.80	6.36	1.39–19.02	6.07
**Protein**	11.5–11.7	9.28–14.86	9.35	11.80	9.95	8.90–11.02	10.21–13.45	11.90–12.82	12.5	9.10	6.23–13.81	11.36

CHO – carbohydrate; NR – not reported. Values are expressed in dry matter.

**Table 2:** Amino acid composition (g/100 g) of sorghum.

Reference	Ala	Arg	Asp	Cys	Glu	Gly	His	Ile	Leu	Lys	Met	Phe	Pro	Ser	Thr	Trp	Tyr	Val
Awadelkareem et al. [40]	8.69–8.70	2.79–3.61	4.83–5.28	NR	17.50–19.57	2.36–3.08	1.75–1.77	3.61–3.72	12.48–13.48	1.57–2.31	1.55–1.88	4.73–5.10	7.70–8.16	3.77–3.85	2.48–2.64	NR	3.72–3.75	4.65–4.74
Khalil et al. [50]	9.0–9.2	3.3	6.5–6.8	1.2	21.6–22	3.0	1.7–1.8	4.0–4.1	13.6–13.8	2.5–2.6	1.3–1.4	5.1–5.2	9.4–10.7	4.3–4.4	3.2	0.9	2.9–3.0	4.8–4.9
Salunkhe et al. [52]	7.34–9.62	3.20–4.68	4.98–6.66	1.94–3.06	23.42–28.12	3.12–4.12	1.46–2.46	3.92–4.86	12.02–14.48	1.42–2.72	1.36–2.34	4.03–5.62	8.92–12.34	3.92–5.66	1.92–2.42	0.49–1.16	2.12–3.62	5.12–6.86
Afify et al. [53]	7.43–8.83	3.58–4.01	6.24–7.06	1.69–2.11	18.45–20.63	2.84–3.05	1.93–2.17	3.49–3.85	11.74–13.56	2.11–2.26	2.73–2.94	4.40–4.98	6.66–8.99	3.49–4.17	2.75–3.21	NR	4.22–4.33	4.22–4.82

Ala – alanine; Arg – arginine; Asp – Aspartic acid; Cys – cysteine; Glu – glutamic acid; Gly – glycine; His – histidine; Ile – isoleucine; Leu – leucine; Lys – lysine; Met – methionine; Phe – phenylalanine; Pro – proline; Ser – serine; Thr- threonine; Trp – tryptophan; Tyr – tyrosine; Val – valine. d.b – dry basis; NR – not reported. Values are expressed in dry matter.

**Table 3:** Mineral composition (mg/kg, d.b) of sorghum.

Reference	Ca	Cd	Co	Cr	Cu	Fe	I	K	Mg	Mn	Na	Ni	P	Pb	Se	Zn
Mabelele et al. [54]	100.1–121.3	NR	NR	NR	1.9–2.4	24.3–40.3	ND	2751–3524	1130–1440	14.2–20.2	20–40	NR	2210–3327	NR	NR	16.2–24.2
Shegro et al. [55]	204.50–447.50	NR	NR	NR	NR	41.17–127.50	NR	1150–2568.75	NR	9.5–23.83	11.5–54.38	NR	1498–3787.25	NR	NR	13.5–34.67
Pontieri et al. [56]	233.84–411.83	9.92–60.54	7.12–15.24	121.59–254.18	NR	39.36–77.03	14.81–212.70	3434.46–6957.67	1454.92–2862.00	8.93–19.44	455.09–840.69	0.46–1.27	2148.60–2963.40	92.62–303.89	2.98–14.13	21.10–47.05
Gerrano et al. [57]	44.57–477.04	NR	NR	NR	NR	13.50–55.13	NR	900–3366	854–1631.17	11.17–20.17	12.50–62.03	NR	2042.19–3775	NR	NR	12–44.83

Ca – calcium; Cd – cadmium; Co – cobalt; Cr – chromium; Cu – copper; Fe – iron; I – iodine; K – potassium; Mg – magnesium; Mn – manganese; Na – sodium; Ni – nickel; P – phosphorus; Pb – lead; Se – selenium; Zn – zinc. NR – not reported. Values are expressed in dry matter.

**Table 4:** Vitamin composition (mg/100 g) of sorghum.

Vitamins	Khalil et al. [50]	Serna-Saldivar and Espinosa-Ramirez [58]	Kulamarva et al. [59]	Ochanda et al. [60]	Saleh et al. [61]
**B_1_**	0.69–0.73	0.45	NR	0.34–0.35	0.38
**B_2_**	0.12–0.14	0.16	0.13	0.15–0.16	0.15
**B_3_**	2.99–3.01	4.88	4.5	4.20–4.55	4.3
**B_5_**	1.55–1.63	NR	NR	NR	NR
**B_6_**	0.40–0.43	0.59	0.47	0.17–0.35	NR
**B_9_**	0.02	0.02	NR	0.02	NR

B_1_ – thiamin; B_2_ – riboflavin; B_3_ – niacin; B_5_ – pantothenic acid; B_6_ – pyridoxin; B_9_ – folic acid. Values are expressed in dry matter.

**Table 5:** Some African sorghum-based fermented food products and microorganisms associated with them.

Product Name	Country/Region	Product Use	Microorganism Identified	Reference
*Aceda*	Sudan	Porridge	Unknown	Eggum et al. [99]; Franz & Holzapfel [100]
*Burukutu*	West Africa	Alcoholic beverage	*Acetobacter* spp., *Candida* spp., *Enterobacter* spp., *Lactobacillus* spp., *Saccharomyces cerevisiae*, *S. chavelieri*, *Leuconostoc mesenteroides*	Kolawole et al. [101]; Eze et al. [102]; Alo et al. [103]; Blandino et al. [104]
*Bushera*	Uganda	Beverage	*Lactobacillus brevis*, *L. delbrueckii*, *L. paracasei*, *L. plantarum*	Marsh et al. [105]; Mwale [106]
*Chibuku*	Zimbabwe	Alcoholic beverage	*Lactobacillus* spp.	Togo et al. [107]; Gadaga et al. [108]
*Dolo*	Burkina Faso/Togo	Alcoholic beverage	*L. delbrueckii*, *L. fermentum*, *L. lactis*, *Pediococcus acidilactici*, *S. cerevisae*	Van der Aa Kühle et al. [109]; Sawadogo-Lingani et al. [110]
*Enturire*	Uganda	Alcoholic beverage	*L. plantarum*, *S. cerevisae*, *Weissela confusa*	Mukisa et al. [111]
*Gowe*	Benin	Porridge	*L. fermentum*, *L. mucosae*	Adinsi et al. [112]; Vieira-Dalodé et al. [113]
*Humulur*	Sudan	Gruel	*Bacillus* spp., *Lactobacillus* spp., yeasts	Adams [114]
*Hussuwa*	Sudan	Porridge	*A. xylinum*, *Gluconobacter oxydans*, *L. fermentum*, *L. saccharolyticum*, *Pediococcus acidilactici*, *S. cerevisiae*	Mwale [106]; Yousif et al. [115]
*Ikigage*	Rwanda	Alcoholic beverage	*Issatchenkia orientalis*, *L. buchneri, L. fermentum*, *Lactobacillus* spp., *S. cerevisiae*	Lyumugabe et al. [116]
*Injera*	Ethiopia	Sourdough/bread	*C. guillermondii*, *Lactobacillus* spp, yeasts	Dandessa et al. [117]
*Kisra*	Sudan	Pancake, flat bread, sourdough	*C. intermedia*, C. *krusei*, *Debrayomyces hansenii, Enterococcus faecium*, *L. amylovorus*, *L. brevis*, *L. confusus*, *L. fermentum*, *Pichia kudriavzevii*	Mohammed et al. [118]; Hamad et al. [119]; Ali & Mustafa [120]
*Khamir*	Sudan	Bread	*L. brevis*, *L. cellobiosus*	Gassem [121]
*Kunun-zaki*	Nigeria	Beverage, breakfast meal	*Ent. faecalis*, *Lactobacillus* spp., *P. pentosaceus*, *W. confusa*	Franz & Holzapfel [100]
*Mahewu*	South Africa	Porridge gruel	*L. brevis*, *L. bulgaricus*, *L. delbruckii, Leuconostoc* spp., *Streptococcus lactis*	Franz & Holzapfel [100]; Hesseltine [122]; Kayitesi et al. [123]
*Mbege*	Tanzania	Beverage	*L. plantarum, Leuc. mesenteroides*, *S. cerevisiae, Schizosaccharomyces pombe*	Odunfa & Oyewole [124]
*Merissa*	Sudan	Alcoholic drink	*Saccharomyces* spp.	Dirar [125,126]
*Nasha*	Sudan	Infant food	*Candida* spp., *Lactobacillus* spp., *S. cerevisiae*, *Strep*. spp.	Graham et al. [127]
*Ogi*	West Africa	Gruel	*L. acidophilus*, *L. agilis*, *L. cellobiosus*, *L. confusus*, *L. murinus*, *L. plantarum*	Graham et al. [127]; Omemu & Bankole [128]
*Ori-ese*	Nigeria	Porridge	*Bacillus subtilis*, *C. tropicalis*, *L. acidophilus*, *L. fermentum*, *L. plantarum*, *Mucor* spp., *Pediococcus* spp., *Penicillium* spp., *S*. *pombe*	Adebayo-Tayo & Needum [129]
*Orubisi*	Tanzania	Alcoholic beverage	LABs, yeasts	Shayo et al. [130]
*Otika*	Nigeria	Alcoholic beverage	*B. cereus, B. subtilis, C. krusei, C. tropicalis, Enterobacter clocae, L. brevis,L. fermentum*, *L. plantarum*, *Leuconostoc mesenteroides, S. cerevisae*	Oriola et al. [131]
*Pito*	Nigeria	Alcoholic beverage	*B. subtillis, Candida* spp., *Geotrichum candidum*, *L. delbrueckii*, *L. fermentum*	Kolawole et al. [101], Sawadogo-Lingani et al. [132]; Ajiboye et al. [133]
*Tella*	Ethiopia	Beverage	*L. pastorianumi*, *S. cerevisae*	Lemi [134]
*Tchapalo*	Ivory Coast	Alcoholic beverage	*L*. *brevis*, *L*. *cellobiosus*, *L*. *coprophilus*, *L*. *fermentum*, *L. hilgardii*, *L*. *plantarum*	Djè et al. [135]; N’guessan et al. [136]
*Tchoukoutou*	Benin	Alcoholic beverage	*L. divergens, L. fermentum, L. fructivorans, S. cerevisae, S. pastorianus, Torulasposa delbrueckii*	Kayodé et al. [137,138]
*Ting*	Botswana, South Africa	Porridge	*L. casei*, *L. coryniformis*, *L. curvatus*, *L. fermentum, L. harbinensis*, *L. parabuchneri*, *L. plantarum*, *L. reuteri*, *L. rhamnosus*	Madoroba et al. [139,140]; Sekwati-Monang & Gänzle [141]
*Thobwa*	Malawi	Alcoholic beverage	Unknown	Nyanzi & Jooste [98]; Matumba et al. [142]
*Uji*	East Africa	Porridge	*L. cellobiosus, L. fermentum, L. plantarum*, *Ped. acidilactici, Ped. pentosaceus*	Blandino et al. [104]
*Umqombothi*	Southern Africa	Beverage	*Lactobacillus* spp.	Katongole [143]
Weaning food	Nigeria	Weaning food	*L. plantarum*, *Ped. acidilactici*, *S. cerevisae*	Wakil & Kazeem [144]

**Table 6:** Some recorded cases of pathogens in African fermented sorghum-based foods.

Food	Safety risk	Probable Source	Reference
Fermented sorghum meal	Food pathogens	*B. cereus*, *Clostridium perfringes*, *Escherichia coli, Listeria monocytogenes*	Kunene et al. [164]
*Hussuwa*	Hygienic risk, antimicrobial resistances, biogenic amines, presence of virulence determinants	*Enterococci*	Yousif et al. [165]
*Gowe*	Cyanogenic compounds, food pathogens, mycotoxins	*E. coli*, Enterobacteriacae, mycotoxigenic fungi	Adinsi et al. [166]
*Ikigage*	Food pathogens	*E. coli, Streptococci*	Lyumugabe et al. [116]
*Mahewu*	Food pathogens	*E. coli*	Simango and Rukure [167]; Nyatoti et al. [168]
*Obushera*	Food pathogens	*E. coli, Staphylococcus*	Byakika et al. [169]
*Ogi*	Mycotoxins	Mycotoxigenic fungi	Adekoya et al. [170]
*Pito*	Mycotoxins	Mycotoxigenic fungi	Ezekiel et al. [148]; Chilaka et al. [171]
*Thobwa*	Mycotoxins	Mycotoxigenic fungi	Matumba et al. [142]
*Ting*	Mycotoxins	Mycotoxigenic fungi	Adebo et al. [153]
